# Supplementary material for: Dynalign II: common secondary structure prediction for RNA homologs with domain insertions
Source: Nucleic Acids Res. 2014 Nov 21;42(22):13939–48. doi: 10.1093/nar/gku1172 (PMC4267632; doi:10.1093/nar/gku1172)
Supplement: SUPPLEMENTARY DATA [file supp_gku1172_nar-02021-z-2014-File012.zip › manual/javadoc/RNAstructure_java_utilities/FileChooser.Types.html]

FileChooser.Types


JavaScript is disabled on your browser.


- Overview
- Package
- Class
- Tree
- Index
- Help

- Prev Class
- Next Class

- Frames
- No Frames

- All Classes

- Summary:
- Nested |
- Enum Constants |
- Field |
- Method

- Detail:
- Enum Constants |
- Field |
- Method


RNAstructure\_java\_utilities

## Enum FileChooser.Types

- java.lang.Object
- - java.lang.Enum<FileChooser.Types>
  - - RNAstructure\_java\_utilities.FileChooser.Types

- All Implemented Interfaces:
  :   Serializable, Comparable<FileChooser.Types>

  Enclosing class:
  :   FileChooser

  ---

    

  ```
  private static enum FileChooser.Types
  extends Enum<FileChooser.Types>
  ```

  An enum that holds the possible values for the file chooser type.

- - ### Enum Constant Summary

    Enum Constants

    Enum Constant and Description

    `OPEN`

    `SAVE`
  - ### Method Summary

    Methods

    | Modifier and Type | Method and Description |
    | --- | --- |
    | `static FileChooser.Types` | `valueOf(String name)` Returns the enum constant of this type with the specified name. |
    | `static FileChooser.Types[]` | `values()` Returns an array containing the constants of this enum type, in the order they are declared. |

    - ### Methods inherited from class java.lang.Enum

      `clone, compareTo, equals, finalize, getDeclaringClass, hashCode, name, ordinal, toString, valueOf`
    - ### Methods inherited from class java.lang.Object

      `getClass, notify, notifyAll, wait, wait, wait`

- - ### Enum Constant Detail


    - #### OPEN

      ```
      public static final FileChooser.Types OPEN
      ```


    - #### SAVE

      ```
      public static final FileChooser.Types SAVE
      ```
  - ### Method Detail


    - #### valueOf

      ```
      public static FileChooser.Types valueOf(String name)
      ```

      Returns the enum constant of this type with the specified name.
      The string must match *exactly* an identifier used to declare an
      enum constant in this type. (Extraneous whitespace characters are
      not permitted.)

      Parameters:
      :   `name` - the name of the enum constant to be returned.

      Returns:
      :   the enum constant with the specified name

      Throws:
      :   `IllegalArgumentException` - if this enum type has no constant
          with the specified name
      :   `NullPointerException` - if the argument is null


    - #### values

      ```
      public static FileChooser.Types[] values()
      ```

      Returns an array containing the constants of this enum type, in
      the order they are declared. This method may be used to iterate
      over the constants as follows:

      ```
      for (FileChooser.Types c : FileChooser.Types.values())
          System.out.println(c);
      ```

      Returns:
      :   an array containing the constants of this enum type, in
          the order they are declared


- Overview
- Package
- Class
- Tree
- Index
- Help

- Prev Class
- Next Class

- Frames
- No Frames

- All Classes

- Summary:
- Nested |
- Enum Constants |
- Field |
- Method

- Detail:
- Enum Constants |
- Field |
- Method
